# Dental Caries, Prevalence and Risk Factors in Patients with Crohn’s Disease

**DOI:** 10.1371/journal.pone.0091059

**Published:** 2014-03-07

**Authors:** Sara Szymanska, Mikael Lördal, Nilminie Rathnayake, Anders Gustafsson, Annsofi Johannsen

**Affiliations:** 1 Department of Dental Medicine, Division of Periodontology, Karolinska Institutet, Huddinge, Sweden; 2 Department of Medicine, Division of Gastroenterology and Hepathology at Karolinska Institutet, Huddinge, Sweden; 3 Stockholm Gastro Center, Sophiahemmet, Stockholm, Sweden; Boston University, United States of America

## Abstract

**Objective:**

The present study tested the hypothesis that patients with Crohn’s disease (CD) have a higher prevalence and risk for caries compared to people without CD.

**Material and Methods:**

Patients with CD were divided into groups; 71 patients (50.7±13.9 years) who had gone through resective intestinal surgery and 79 patients (42.0±14.4 years) who had not. The patients were compared to 75 controls (48.6±13.4 years) regarding DMF-T and DMF-S, *Lactobacilli* (LB), *Streptococcus mutans* (SM), salivary flow and dental plaque. Statistical methods including ANOVA or Chi-square test for calculation of demographic differences between groups, analysis of covariance (ANCOVA) to compare the clinical variable and Post hoc analyses were done with Fischers Least Significant Difference test or Chi-square. Non-parametric Spearman’s correlation matrix coefficient was estimated between clinical variables and disease duration.

**Results:**

CD patients who had been subjected to resective surgery had a higher DMF-S score (50.7 *versus* 36.5; *p* = 0.01) compared to the control group after adjusting for age, gender and smoking. These patients had higher counts of SM (1.5 *versus* 0.9; *p* = 0.04) and LB (10000.0 *versus* 1000.0; *p* = 0.01), and more dental plaque (53.7 *versus* 22.6; *p* = 0.001). CD patients reported a more frequent consumption of sweetened drinks between meals compared to controls (*p* = 0.001).

**Conclusions:**

The present study shows that patients with CD who had undergone resective surgery had a higher DMFs score, and higher salivary counts of *Lactobacilli and Streptococcus mutans* compared to the control group.

## Introduction

Crohn’s disease (CD) is a granulomatous chronic inflammatory disease that can affect any region of the gastrointestinal tract, although it is usually localized to the small intestine and colon [1]. First described by Crohn et al. [2], the diagnosis of CD is based on clinical signs, endoscopy, histology, radiographic and/or biochemical findings [3]. CD shows episodes of disease activity, so called flares and asymptomatic intervals, or remissions [4]. This often leads to recurrent episodes of illness during which treatment with drugs and sometimes surgery is required to achieve symptomatic remission [5]. The aetiology of CD is unknown, however, studies have linked a possible genetic association [6]. In addition, a link between the microbiota and the lining of the gut mucosa has also been proposed as possible aetiological environmental factors [1], [7]. The incidences of CD differ depending on geographical region. North America and the northern part of Europe have the highest incidence [8], [9]. The prevalence of CD in adults in the US is 201 per 10^5^ people [8].

Established risk factors for dental caries are increased number of *Lactobacilli* (LB) and *Steptococcus mutans* (SM), decreased salivary flow, insufficient oral hygiene, poor dietary habits including increased sugar consumption, as well as socioeconomic factors [10], [11].

Earlier studies have reported higher caries prevalence in CD patients compared to controls [12]. Indeed CD patients exhibited higher levels of LB and SM [13], [14]. Brito et al. [15] reported an increased mean value in the decayed, missed, filled teeth (DMF-T) score amongst CD patients compared to controls. Furthermore, a study from the US reported that patients with CD had more caries, increased mouth dryness and visited the dentist more often [16]. A questionnaire study from our group showed that patients with CD perceived their oral health to be poor and reported significantly more mouth-related problems and a greater requirement for dental treatment compared to a control group [17]. Thus, the aim of present study was to test the hypothesis that patients with CD have a higher prevalence and risk for caries compared to people without CD.

## Materials and Methods

Patients with an established diagnosis of CD, according to Lennard-Jones criteria [18], attending the outpatient clinic at the Department of Gastroenterology and Hepathology at Karolinska Hospital were invited to participate in the study. Out of 309 patients that were consecutively asked to participate, 150 patients with CD were enrolled (73 females and 77 males), aged 18–77 years, between September 2008 and June 2010.

The control group were selected from 181 individuals that were randomly recruited through National Statistics Organization (SCB) in Sweden. 75 individuals (45 females and 30 males) accepted to participate in the study, aged 18–74 years. The selection of the control group was structured to achieve the same age and gender distribution as the patient group. All participants were living in Huddinge community of Stockholm and had no history of CD.

Ethical approval was obtained from the Karolinska Institutet Ethical Research Board (ref. nr.2007/2∶11, 2009/1953-32), as well as oral and written informed consent from each participant before commencing the investigations.

### Questionnaire

All participants completed a questionnaire that covered demographic data including age, gender, income, education level, medical history, medications, and smoking habits. Most of the questions in the questionnaire were of the multiple-chose type. The questions were based on the questionnaire from our earlier study ([17]). Smoking habits were reported as current smokers, former-smokers and never smokers, the response alternatives were yes or no. Questions concerning oral hygiene practice included frequency of tooth brushing, interproximal cleaning, and visits to the dentist and/or dental hygienist. Dental health was also registered by enquiring if they had reported any toothache, problems with oral ulcerations, dry mouth and bad breath during the last 12 months. The response alternatives to these questions were yes or no. In addition, all participants were asked about eating habits including frequency of meals and consumption of sweetened drinks between meals. The patients with CD were also asked how long they have had their disease, and if they had undergone surgical procedure.

### Clinical Examination

Two investigators (SS/193 subjects, NR/32subjects) examined the participants. Prior to the clinical examination, an inter and intra calibration between the two examiners was conducted. Three participants (from CD group) were examined by both investigators to reach an agreement. In addition, repeated measurements within two patients were done by the same examiners.

The clinical examinations were conducted after saliva sampling, registration of the number of teeth and assessment of dental plaque (Visible Plaque Index, VPI) at six sites on all present teeth, excluding third molars.

### Caries Registration

Caries was assessed by World Health Organization methods [19] and expressed by decayed, missing, or filled tooth (DMF-T) and surface index (DMF-S) in each person. The diagnosis was based on a clinical inspection and on x-rays. The diagnosis was based on a clinical inspection, visually and tactically with an examination probe and on radiographs. Clinically, caries was recorded when a lesion had a cavity, undermined enamel or an obviously softened surface, and the probe became stuck by using light pressure. Filled surfaces with caries were registered as both decayed and filled.

The radiographic examination was done with 4 bitewing radiographs. All tooth surfaces that could not be evaluated clinically were evaluated on the radiographs. Dental caries on the radiographs was recorded if the lesion reached the dentine.

The examiner of the radiographic images was blinded. An intra-examiner measurement analysis was performed in 10% of the patients and controls (randomly selected), and the measurements reached identical results in 89% of the cases.

### Saliva Sampling

To avoid contamination of the oral cavity as a result of food intake or smoking, the subjects were instructed not to eat, drink, smoke or brush their teeth one hour before sampling. Unstimulated whole saliva and saliva stimulated by chewing paraffin wax was collected during a five minute period. Salivary flow rates were measured in millilitres per minute immediately after collection.

### Analyses of *Lactobacilli* and *Streptococcus Mutans*

Salivary LB and SM counts were measured using Dentocult-LB Orion Diagnostica and Dentocult-SM Orion Diagnostica, according to the manufacturer’s instructions. The salivary level of *Lactobacilli* is expressed as bacteria per mL and level of *S. mutans* in an arbitrary unit, 0–3. The numbers 0–1 represents less than 100 000 colony forming units (CFU)/mL, 2 represents 100 000–1000 000 CFU/mL and 3 represents more than 1000 000 CFU/mL.

### Statistical Analysis

Analyses of the data were performed using the software package PASW Statistics 18 (PASW Inc., Chicago, IL, USA). The significance of the demographic differences (Table 1) between patients and controls were calculated with ANOVA, variables with only two factors were calculated with Chi-square test. Post hoc analyses were done with Fischers Least Significant Difference test or Chi-square. Analysis of covariance (ANCOVA) to control for age, gender and smoking were performed to compare the clinical variables between the groups, post hoc analyses were done with Fischers Least Significant Difference test (Table 2). P-values of 0.05 or below were considered significant. Non-parametric data were normalised with a logarithmation. Non-parametric Spearman’s correlation matrix coefficient was estimated between clinical variables and disease duration.

**Table 1:** Demographic data, mean (SD) and percentages (number) for the CD patients (who had not and had undergone resective surgery) and the controls.

Variable	Control groupn = 75	*p* 1	CD PatientsNo resective surgery n = 79	*p* 2	CD PatientsResective surgeryn = 71	*p 3*
**Gender** F/M (n)	46/29		39/40		38/33	NS
**Age** (mean ± SD)	48.6 (±13.4)	0.003	42.0 (±14.4)	NS	50.7 (±13.9)	0.001
**Weight,** kg	74.4 (±13.0)		70.5 (±16.6)		74.3 (±14.4)	NS
**Length** cm	170.8 (±15.8)		170.8 (±9.8)		170.0 (±8.6)	NS
**Smokers** % (n)	6 (5)	0.030	19 (15)	0.005	25 (17)	0.012
**Former smokers** % (n)	46 (33)		41 (30)		37 (25)	NS
**High blood pressure** % (n)	9 (5)		19 (15)		17 (12)	NS
**Cardiovascular disease** % (n)	7 (5)		3 (2)		3 (2)	NS
**Rheumatoid arthritis** % (n)	5 (4)		13 (10)		7 (5)	NS
**Kidney disease** % (n)	4 (3)		5 (4)		7 (5)	NS
**Diabetes** % (n)	0 (0)		4 (3)		6 (4)	NS
**Cancer** % (n)	1.3 (1)		4(3)		6 (4)	NS
**Osteoporosis** % (n)	3 (2)		4 (3)		6 (4)	NS
**Lung disease** % (n)	1 (1)		8 (6)		7 (5)	NS
**Asthma** % (n)	21 (16)		29 (22)		23 (16)	NS
**Use medication regularly** % (n)	32 (23)	0.001	87 (66)	0.001	81 (54)	0.001
**Behavior characteristic**						
*No Fibrostenotic/Penetrating,*% (n)			71 (56)		35 (25)	
*Fibrostenotic* % (n)			23 (18)		51 (36)	
*Penetrating* % (n)			6 (5)		14 (10)	
**CD duration** (mean ± SD)			8 (8)		22 (14)	
**Meal frequency**	4.06 (1.5)		4.01 (1.4)		3.97 (1.5)	
**Sweetened drinks** % (n)	26 (20)	0.043	43 (34)	0.001	61 (43)	0.001
**Toothache** % (n)	14 (11)		24 (18)		19 (13)	NS
**Dry mouth** % (n)	11 (8)	0.001	38 (30)	0.011	29 (20)	0.001
**Bad breath** % (n)	12 (9)	0.003	33 (26)	NS	21 (15)	0.008
**Ulcers** % (n)	23 (17)		27 (21)		23 (16)	NS

*p* 1 indicates significance of the difference between controls and CD patients who had not undergone rescective surgery.

*p 2* indicates significance of the difference between controls and CD patients who had undergone rescective surgery.

*p 3* indicates significance of the difference between the 3 groups.

Significances were calculated with ANOVA and Chi-square test. Post doc analyses were done with Fischers’ least significant difference test. NS = Not significant.

The difference in CD duration calculated with unpaired Student’s t-test.

**Table 2:** Mean (SD) for the DMF-T/DMF-S index, *Steptococcus mutans*, volume of stimulated - and unstimulated saliva, the amount of dental plaque, and median (interquartile range) for *Lactobacilli*, in CD patients (who had not and had undergone resective surgery) and controls.

Variable	Control groupn = 75	*p* 1	CD PatientsNo resective surgeryn = 79	*p* 2	CD PatientsResective surgeryn = 71	*p 3*
DMF-T	13.1 (6.5)		11.2 (7.1)		15.5 (8.3)	NS
DT	1.1 (2)		1.8 (2.9)		2.2 (3.2)	NS
MT	1.8 (3.3)		1.8 (2.9)		2.7 (4.1)	NS
FT	10.12 (5.4)		8.0 (5.4)		10.6 (6.4)	NS
DMF-S	36.5 (26.9)	NS	33.1 (28.6)	0.004	50.7 (36.2)	0.014
DS	1.5 (2.2)		2.7 (5.9)		3.6 (7.6)	NS
MS	8.5 (15.4)		8.9 (13.7)		13.3 (19.9)	NS
FS	26.5 (19.4)		22.6 (19.1)		33.7 (24.5)	NS
*Modifiers*						
*Lactobacilli*Bacteria/mL	1000 (1000)	0.008	10000 (99000)	0.012	10000 (99000)	0.011
*Steptococcus mutans* *Arbitrary unit*	0.9 (0.9)	NS	1.5 (0.9)	0.04	1.5 (1.1)	0.016
Stimulated saliva ml/min	2.0 (0.8)		2.2 (0.9)		2.0 (0.9)	NS
Unstimulated saliva ml/min	0.65 (0)		0.62 (0.4)		0.56(0)	NS
VPI	22.6 (22.1)	0.001	45.3 (25.9)	0.001	53.7 (29.2)	0.001

DMF-T = decayed, missed, filled teeth, DT = decayed teeth, MT = missing teeth, FT = filled teeth, DMF-S = decayed, missing, filled surface, DS = decayed surface, MS = missing surface, FS = filled surface, VPI = Visible Plaque index.

*p* 1 indicates statistical significance of the difference between controls and CD patients who had not undergone rescective surgery.

*p* 2 indicates statistical significance of the difference between controls and CD patients who had undergone rescective surgery.

*p* 3 indicates significances difference between all three groups.

Significances calculated with ANCOVA (analysis of covariance), adjusted for age, gender and smoking. Post hoc analyses done with Fischer’s least significant difference test. NS = Not significant.

### Study Population

The number of patients with CD (n = 150) and the number of controls (n = 75) was chosen to allow us to observe differences in oral health of 10% of the population with a power of over 90%. Smaller differences were not considered clinically relevant. The power calculation was based on the differences reported by Brito et al. [15]. The power calculation revealed that 150 CD patients and 75 controls would provide 80% power to detect a difference in means of DMFT of 2.8 between the groups (22%), assuming that the common standard deviation is 7.0, with a 0.05 two-sided significance level.

## Results

An initial analysis of the data showed that there were two distinct subgroups in the patient’s population, those who had undergone resective surgery (RS) and those who had not (NRS). For this reason, the two groups were compared separately to the control group. Demographic data for all CD patients and the control group are presented in Table 1. There was a significant difference in age (*p* = 0.001) and duration of CD (*p* = 0.01) between the RS and NRS group. There were no significant differences between the groups regarding marital status, income and education.

There was no difference between the groups regarding frequency of meals. However, patients with CD reported significantly more frequent intake of sweetened drinks between the meals, such as soft drinks, compared to the control group (Table 1). There were significant differences between the groups regarding dry mouth and bad breath during the last 12 months as determined by a self-administrated questionnaire (Table 1).

Table 2 shows the clinical variables amongst the three groups, using ANCOVA and adjusted for age, gender and smoking. The RS group had a significantly higher DMF-S score. The difference was most pronounced regarding the number of filled surfaces (FS), although the difference did not reach statistical significance. The RS group had also a higher DMF-T score but the difference did not reach statistical significance (*p* = 0.06).

Both CD groups had significantly higher levels of LB and amounts of dental plaque compared to the control group. In addition, the RS group had more of SM compared to the control group (Table 2).

The results showed a weak positive correlation between disease duration and DMF-S (r = 0.374, *p = *0.01), missing surface (MS) (r = 0.272, *p = *0.05), and filled surface (FS) (r = 0.424, *p = *0.01) in the RS group. These correlations could not be found in the NRS group.

When comparing the frequency of visits to the dentist and dental hygienist amongst the patients and controls, there were no significant differences. Oral hygiene habits did not differ regarding frequency of tooth brushing, and the use of approximal aids between the two groups (no data shown).

### Gender Differences

Men in the CD group had significantly more decayed teeth (DT) (2.5±3.7 vs. 1.5±2.1, *p* = 0.05), and decayed surface (DS) (4.3±8.6 vs. 2.1±4.1, *p* = 0.05) compared to women. The mean percentages of dental plaque were higher in men compared to women in the CD group (56.4±27.1 vs. 42.4±26.1, *p* = 0.005). There were no gender differences regarding dental caries assessment and the percentages of dental plaque in the control group.

### Non-responders

Of those invited to participate 159 patients in the CD group and 106 individuals in the control group declined, albeit the reason was not asked. A statistical comparison between responders and non-responders showed no significant differences regarding age and gender.

## Discussion

The present study revealed that CD patients who had undergone resective surgery had higher DMFs scores compared to patients without CD after adjusting for age, gender and smoking. More caries in CD patients have been shown by some previous studies [13], [15], [20]. Conversely, Grössner - Schreiber et al. [21] did not report any differences in the DMF-S index between patients with inflammatory bowel disease (IBD), but described a significantly higher prevalence of dentine caries amongst patients with IBD compared to the control group. An explanation for this discrepancy might be the different study groups, since patients with IBD include both CD and ulcerative colitis. The present study found more dental plaque in both CD patient groups, as compared to the controls, which could influence the prevalence of caries and is in agreement with a recent study by Habashneh et al. [22].

In this study both patient groups consumed more sweetened drinks between meals compared to the controls. This is in agreement with earlier epidemiological studies showing that patients with CD consumed larger amounts of highly refined carbohydrates, such as candy and/or soft drinks that are associated with increased dental caries risk [20], [23]. Furthermore, several studies have shown that CD patients have a higher sugar intake compared to healthy controls even before the onset of disease [23], [24]. Schütz et al. [20] demonstrated higher caries prevalence, higher sugar intake as well as lower zinc plasma levels in CD patients, but they were unable to relate these changes to each other. Zinc deficiency can influence sweet taste perception in patients with CD [25]. The mechanism of zinc deficiency remains unclear, and there are contradictory results as to whether in fact there are decreased plasma levels of zinc in CD patients compared to controls [25], [26]. Our study did not show any differences in meal frequency between patients and controls.

The levels of LB and SM were higher in CD patients compared to the control group, which is in line with Sundh et al. [14]. Furthermore, several studies have found that these bacteria are involved in caries activity although it must be taken into consideration that species variations exist for these bacteria, for review see Takahashi and Nyvad [27]. The involvement of bacteria in the caries process is complex and remains unclear.

The biological agent, tumor necrosis factor alpha (TNFα) inhibitor, has an anti-inflammatory effect and is used when other medical treatment for Crohn’s disease have failed [28]. ΤΝFα-inhibitor is currently regarded as part of the treatment of choice for CD when other therapies have shown to be ineffective, but it has only been available during the last decade. This approach has reduced the need for resective surgery. In our study population very few patients had received the anti-TNF treatment. Thus, this treatment had no influence on the comparison between patient’s undergone surgery or not. In the current study, patients that had undergone surgery had a higher DMFs score compared to the control group, while there were no difference between the patients who had not undergone surgery and the controls. This difference remains also after consideration for age and gender. Disease duration showed a weak but significant correlation with DMFs in resective group but not in the non-surgery group. The reason for this difference between the patient groups could be that the surgery group have had a more severe disease leading to a need for surgery. We found no differences in eating habits between the two patient groups. Another explanation could be that over the years of suffering with the disease prior to the resective surgery they experienced problems with their oral health status and many caries restorations were performed. It is well established that one of the risk factors for caries are old restorations and in turn might increase the risk for more cavities and oral health problems in this group.

Interestingly, in the present study, the men in the patient group had a significantly higher prevalence of dental plaque compared to the women in the same group, even if there are individual variations, whereas there were no gender differences in the control group. To date, no study exists considering gender perspective and oral health in patients with CD. The clinical significance of the present study is that patients with CD who had undergone RS, particularly men, seem to be in a need for individual caries prophylaxis.

The high numbers of drop-outs from the current study need consideration. All patients were contacted by phone and some of those who didn’t want to participate in the study mentioned they were sick, or they had visited the dentistry recently and, some person gave no reason.

Living with CD, means coping with a lifelong condition sometimes requiring significant lifestyles adjustments that might influence oral hygiene behaviours and dietary habits. Keefer et al. [29] showed that patients with IBD who received behavioural interventions seemed to alter the disease course and improve their quality of life. Therefore it is important that patients with CD are instructed about the relationship between their disease and the increased risk for caries. In the clinical practice these patients must be informed about the consequences of altered dietary habits and the importance of prevention. Further research should focus on developing individual preventive programmes tailored to their needs.

The present study shows that patients with CD who had undergone resective surgery had a higher DMFs score, and higher salivary counts of *Lactobacilli and Streptococcus mutans* compared to the control group.
